# A Quantitative Risk Assessment Model for *Listeria monocytogenes* in Non-Ready-to-Eat Frozen Vegetables

**DOI:** 10.3390/foods13223610

**Published:** 2024-11-12

**Authors:** Ursula Gonzales-Barron, Régis Pouillot, Juliana De Oliveira Mota, Akio Hasegawa, Ana Allende, Qingli Dong, Matthew J. Stasiewicz, Jovana Kovacevic, Vasco Cadavez, Laurent Guillier, Moez Sanaa

**Affiliations:** 1 Centro de Investigação de Montanha (CIMO), Instituto Politécnico de Bragança, Campus de Santa Apolónia, 5300-253 Bragança, Portugal; vcadavez@ipb.pt; 2 Laboratório Para a Sustentabilidade e Tecnologia em Regiões de Montanha, Instituto Politécnico de Bragança, Campus de Santa Apolónia, 5300-253 Bragança, Portugal; 3 Independent Researcher, 18 rue Mohamed Al Ghazi, Rabat 10170, Morocco; rpouillot.work@gmail.com; 4 Nutrition and Food Safety Department, World Health Organization, 1202 Geneva, Switzerland; deju@who.int (J.D.O.M.); hasegawaa@who.int (A.H.); 5 Centro de Edafología y Biología Aplicada del Segura, Consejo Superior de Investigaciones Científicas (CEBAS-CSIC), Campus de Espinardo, 25, 30100 Murcia, Spain; aallende@cebas.csic.es; 6 School of Health Science and Engineering, University of Shanghai for Science and Technology, Shanghai 200093, China; qdong@usst.edu.cn; 7 Department of Food Science and Human Nutrition, University of Illinois at Urbana-Champaign, Champaign, IL 61801, USA; mstasie@illinois.edu; 8 Food Innovation Center, Oregon State University, Portland, OR 97209, USA; jovana.kovacevic@oregonstate.edu; 9 Risk Assessment Department, French Agency for Food, Environmental and Occupational Health & Safety (Anses), 14 rue Pierre et Marie Curie Maisons-Alfort, 94701 Maisons-Alfort, France; laurent.guillier@anses.fr

**Keywords:** frozen produce, blanched vegetables, listeriosis, exposure assessment, simulation

## Abstract

A quantitative risk assessment (QRA) model was developed to evaluate the risk of invasive listeriosis from the consumption of non-ready-to-eat (non-RTE) frozen vegetables. On a lot basis, the QRA model simulates *Listeria monocytogenes* concentration and prevalence in a “Processing module” that comprises blanching, potential recontamination and packaging, any post-packaging inactivation treatment, and within-lot end-product testing and in a subsequent “Consumer’s handling module” that encompasses portioning of frozen vegetables, defrosting, and cooking. Based on available published data, the model was coded in nine sequential R functions designed to assess the effectiveness of blanching, the improvement in processing environment hygiene, the implementation of sampling schemes at the end of processing, and improved consumer instructions on the product’s package. In a reference scenario, the model estimated that 9.4% of 500 g packages of frozen vegetables would be contaminated, although at mean levels lower than 10 CFU/g, and assuming that 20% of the portions of frozen vegetables would be left to thaw at room temperature for 2 h, the lot-level mean risk of listeriosis in the susceptible population would be 2.935 × 10^−14^ (median 5.446 × 10^−15^) for uncooked 50 g servings and 2.765 × 10^−17^ (median 5.184 × 10^−18^) for cooked 50 g servings. Analysis of selected scenarios suggested that not cooking the non-RTE product contributes to the risk to a greater extent than the level of contamination in the incoming raw vegetables, the latter in turn being more influential than the level of contamination in the processing environment. The QRA model is freely available as an R package with full documentation and can be used as a tool to inform the consideration of strengthened risk management measures in view of the current changes in consumer behavior and new diet trends.

## 1. Introduction

Recent listeriosis outbreaks have prompted concerns about the risks associated with the consumption of non-RTE frozen vegetables. Between 2013 and 2015, a multi-state outbreak of listeriosis took place in the United States, involving nine cases with three fatalities. Whole-genome sequencing (WGS) analysis demonstrated that an *L. monocytogenes* strain isolated from frozen corn was genetically related to eight bacterial isolates from eight human cases, whereas an isolate from frozen peas from the same manufacturer yielded a different *L. monocytogenes* that was indistinguishable to that from an additional human case. It was concluded that the contamination source of this outbreak was the processing environment [1]. Later on, between 2015 and 2018, an outbreak of invasive *L. monocytogenes* infections occurred across Austria, Denmark, Finland, Sweden, and the UK, involving 53 cases and 10 fatalities. The source of the outbreak was traced to sweet corn grown and quick-frozen in Hungary. WGS identified the outbreak strain (*L. monocytogenes* 4b, multi-locus sequence type ST 6) in various frozen food samples and environmental swabs from a plant where vegetables were processed and frozen. Environmental contamination was indicated as the source of the persistence of the strain causing the outbreak from 2015 to 2018 [2]. The processing of these vegetables included a blanching step before freezing [3,4].

The European Commission [5] and the Codex Alimentarius [6] have established microbiological criteria for *L. monocytogenes* in RTE foods depending on whether the food product permits growth under storage conditions throughout its shelf-life. For RTE foods that support the growth of *L. monocytogenes*, the criterion of absence in 25 g applies to five samples per batch. For RTE foods that do not support the growth of *L. monocytogenes*, the microbiological limit for acceptable batches is 100 CFU/g in five samples at the end of shelf life. While *L. monocytogenes* does not grow in frozen foods and therefore, a threshold of 100 CFU/g would be accepted for these products, such criterion might not be enough to control the production process if defrosted frozen vegetables are stored for a certain period, even at refrigeration temperatures, before consumption [7]. The potential growth of *L. monocytogenes*, initially present in frozen vegetables even at very low concentrations, would then represent a more serious public health risk. On the other hand, non-RTE frozen foods have no regulatory limit when considering the number of *L. monocytogenes* in the product, and these were the kinds of foods causing the outbreaks mentioned above. According to Zoellner et al. [8], outbreak investigations showed that alternative (non-intended) preparations of non-RTE frozen vegetables were the root of the problem, such as defrosting and direct use in salads, or as ingredients in other RTE products subsequently sold to consumers without undergoing any treatment to reduce the level of pathogens.

In this respect, two quantitative risk assessment (QRA) models for non-RTE frozen vegetables have been published: Zoellner et al. [8] and EFSA [7]. EFSA’s model [7] focused only on the home preparation module and considered cooking as the most important control measure to reduce risk. They estimated that decreasing the proportion of uncooked servings from 23% (baseline) to 4% (best-case scenario) reduces the predicted listeriosis cases per year from 1.62 to 0.041 in elderly females in the European Union. The EFSA model [7] concluded that blanched frozen vegetables conveyed the lowest risk of listeriosis in comparison to sausage, hot-smoked fish, pate, gravad fish, cooked meat, and cold-smoked fish. The model of Zoellner et al. [8] (made available as the FFLLoRA tool) represented not only the consumer’s module but also considered two single features of the processing stage: within-batch testing and batch size. According to the FFLLoRA tool, the median risk of illnesses per batch of frozen vegetables is inferior to 10^−16^ when at least half of the servings are properly cooked. They further stressed the importance of cooking through sensitivity analysis, as it showed that cooking the vegetables serving (Spearman rank coefficient: *r* = −0.87) and log reduction due to proper cooking (*r* = −0.48) were the most important drivers determining the exposure dose of *L. monocytogenes* consumed per serving.

Whereas these two QRA models provided valuable insights into the impact of consumer preparation and handling, the contributions to the final risk from sources and factors inherent to the processing stage remained unknown. This understanding is particularly important since *L. monocytogenes* can contaminate vegetables post-blanching [3,4,9]. Within this context, the main objective of this work was to construct a QRA model of longer scope for non-RTE frozen vegetables, able to represent the inactivation, recontamination, and potential growth of *L. monocytogenes* in non-RTE frozen vegetables from processing to consumption. Widely based on published data, the model was structured to be able to assess the effectiveness of the following treatments/control measures: blanching, any additional inactivation step post-packaging, reducing contamination in the processing facility environment, sampling schemes at the end of processing, better labeling and/or consumer education (represented by instructions on proper storage, defrosting and cooking practices at home), in line with the recent Expert Panel recommendation of the Joint FAO/WHO Expert meeting on microbiological risk assessment [10]. The functionality of the QRA model proposed was illustrated by a reference scenario, supported by literature data, and by the development of what-if scenario analysis.

## 2. Materials and Methods

### 2.1. Exposure Assessment

The exposure assessment model of non-RTE frozen vegetables comprises two modules: the “Processing module”, which represents blanching, potential recontamination and packaging, post-packaging inactivation treatment, and within-batch testing; and the “Consumer’s module”, which encompasses portioning, defrosting, and cooking. Each stage was coded as a function estimating stochastically the microbial prevalence and number after a process of microbial growth, death, partitioning, removal, or cross-contamination [11], according to the stage’s purpose. Table 1 summarizes the modules, the sequence of stages and processes they consist of, the assumption and data sources employed, and the corresponding programmed functions.

#### 2.1.1. Contaminated Lots of Vegetables Pre-Blanching

**Data and assumptions:**

The distributions for prevalence and concentration of *L. monocytogenes* in pre-blanched vegetables were modeled from published data. However, since data on pre-blanched vegetables are very scarce, *L. monocytogenes* prevalence in fresh and minimally processed vegetables was instead used. Such data were extracted from the Pathogens-in-Foods database [12]. The lot-to-lot variability in the prevalence of *L. monocytogenes* in pre-blanched vegetables was assumed to follow a Beta distribution with parameters *α* and *β*, which were determined using the surveys’ data compiled in Table 2. Every observed sampling outcome (*s_j_* positive samples out of *n_j_* total samples) was assumed to originate from the *j*-th lot of pre-blanched vegetables (*j* = 1, 2, … *N*), where *N* is the number of lots sampled (*N* = 21, according to Table 2). The sampling outcomes *s_j_* are considered as realizations of a binomial distribution (*n_j_*, *p_j_*), where *p_j_* is the unobservable prevalence of the lot *j*. The values of *α* and *β*, the beta distribution parameters that describe the between-lot prevalence, were estimated by Markov Chain Monte Carlo (MCMC) simulation as the means of posterior distributions in a Bayesian framework. The model was expressed as

(1) $$s_{j}\sim Binomial\left(n_{j},p_{j}\right)\;p_{j}\sim Beta\left(\alpha ,\beta \right)$$

with non-informed priors for *α* and *β* assumed to be Gamma distributions with shape and rate parameters both set to 0.01. The parameters were derived from 20,000 iterations and a burn-in period of 5000 iterations. The mean value for *α* was estimated at 0.5112 (median: 0.4816; standard deviation: 0.1962; 95% credibility interval: 0.220–0.985), and the mean value for *β* at 9.959 (median: 9.100; standard deviation: 4.915; 95% credibility interval: 2.971–21.92).

**Table 1:** Sequence of stages, microbial processes represented, data sources, assumptions, and corresponding functions coded in R for the construction of the exposure assessment model of *Listeria monocytogenes* (LM) in non-RTE frozen vegetables.

Module	Stage	Microbial Process	Assumptions	References	Function in R
Processing	Generation of contaminated lots pre-blanching	None	LM prevalence in pre-blanched vegetables is assumed to be comparable to the prevalence found in minimally processed vegetables and fresh whole vegetables sampled at the packinghouse or at retail. LM concentration in pre-blanched vegetables is assumed to be comparable to the concentrations found in freshly harvested vegetables and vegetables sold at retail.	Badosa et al. [13], Cardamone et al. [14], Carp-Carare et al. [15], Cetinkaya et al. [16], De Giusti et al. [17], Gianfranceschi et al. [18], Kokkinakis et al. [19], Lika et al. [20], Losio et al. [21], Magdovitz et al. [22], Moreno et al. [23], Pianetti et al. [24], Vojkovska et al. [25], Wagner et al. [26] Jeyaletchumi et al. [27], Kuan et al. [28]	Lot2LotGen()
	Blanching	Inactivation	LM during blanching is assumed to follow the kinetics of a cocktail of N-7004 (Scott A, serotype 4b), N-7285 (serotype 1/2a), N-7298 (serotype 1/2b), and N-7017 (Murray B, serotype 4b) LM inoculated in broccoli, mushroom, onions, peas, and pepper.	Mazzotta [29]	fvBlanching()
	Freezing and packaging	Cross-contamination and partitioning	LM can contaminate the bulk of vegetables post-blanching from equipment such as feeders, slicing machines, transporters, conveyor belts, and freezing tunnels. If the contamination event takes place, LM cells are transferred to the bulk according to a transfer coefficient from stainless steel to vegetables. LM is assumed to be moderately clustered in the bulk of frozen vegetables from a lot.	Truchado et al. [3] Hoelzer et al. [30] Nauta [31]	fvPartitioningCC()
	Post-packaging treatment	Reduction	Generic inactivation step post-packaging. Assumption of independent, equal probability of inactivation.	Nauta [32]	fvReductionPostpack()
	Within-lot testing	None	At a given probability, a lot of frozen vegetables can be subjected to sampling and testing according to a two-class or three-class microbiological sampling plan.	-	fvTesting()
Consumer’s preparation	Portioning	Partitioning	The consumer is assumed to take a portion of frozen vegetables from the pack. LM cells present in a contaminated pack are assumed to be moderately clustered within the package.	Nauta [31]	fvPortioning()
	Defrosting	Growth	The consumer may defrost the frozen vegetables in the fridge, microwave, or at room temperature before cooking or consumption. LM is assumed to grow according to a log-linear model without a lag phase, based on an exponential growth rate at 5 °C for heat-treated vegetables that distribute as a lognormal distribution.	Zoellner et al. [8] EFSA [7]	fvDefrost()
	Cooking	Reduction	There is a probability that the consumer uses the non-RTE frozen vegetables in a non-intended manner, such as through direct (uncooked) consumption in salads, smoothies, etc. For cooked vegetables, different heat treatment intensities can be applied by consumers, varying from very strong heat treatments (i.e., fully cooking) to light heat treatments (i.e., microwave heating). Therefore, there is a variability in the effect of cooking.	Willis et al. [33], FSAI [34] EFSA [7]	fvCooking()

Thus, the prevalence of *L. monocytogenes* in a lot of pre-blanched vegetables was sampled from a Beta (0.5112, 9.959) distribution. For reference, such a distribution has a mean of 5.13% and a 95% variability interval of 0.06–28.48%.

Available data on the concentration of *L. monocytogenes* in pre-blanched (or fresh) vegetables were very scarce. In Malaysia, Jeyaletchumi et al. [27] found 3.0 MPN/g in a sample of freshly harvested carrots and 3.6 MPN/g in a sample of freshly harvested yardlong beans, whereas Kuan et al. [28] determined 3.0 MPN/g in one sample of carrots at retail. Non-published data of *L. monocytogenes* in green beans sold in markets from our laboratory were added to the data set: 21.0, 36.0, and 75.0 MPN/g. Because the six observations available were obtained from the MPN based on serial dilution analysis, the statistical model of the MPN was exploited in a Bayesian framework to model the distribution of the total variability in the log_10_ concentration of *L. monocytogenes*.

A conditional probability of observing the tube counts given the unknown *L. monocytogenes* concentration in a contaminated vegetable unit was built from the MPN triplets of every vegetable sample tested. According to a three-dilution (0.1, 0.01, 0.001) MPN table, the tube result triplets (*x*_1_-*x*_2_-*x*_3_) of the contaminated vegetable samples were 1-0-0 (3.6 MPN/g); 2-2-0 (21.0 MPN/g); 2-3-1 (36.0 MPN/g); 3-1-1 (75.0 MPN/g); and for the two incidences of 3 MPN/g, the triplets 0-0-1 and 0-1-0 were assumed.

The MPN technique assumes that the microorganisms from a sample at concentration *λ* (cells per gram) are well mixed in the homogenate and that the aliquots taken from it contain a Poisson (*λ* × *df_i_*) distributed number of microorganisms, where *df_i_* is the dilution factor or the amount of sample in g per ml in the tube corresponding to the dilution *i*. The probability *p_i_* of having a positive test tube (at least one cell present in the tube) belonging to the dilution *i* arises from the Poisson process, whereas out of *n_i_* serial dilution analysis tubes (or trials), the numbers of positive tubes (*x_i_* or successes) follow a binomial distribution. For our data, the number of dilutions is three (*i* = 3), and the number of analysis tubes per dilution is three (*n*_1_ = *n*_2_ = *n*_3_ = 3). Thus, the mean $\mu_{C_{0}}$ and the standard deviation $\sigma_{C_{0}}$ of the normal distribution characterizing the total variability in the log_10_ concentration of *L. monocytogenes* in pre-blanched vegetables (*C*_0_) were estimated in a Bayesian framework through MCMC simulation by adjusting the MPN triplets ((*x*_1j_, x_2j_, *x*_3*j*_) for *j* = 6 observations) to the model

(2) $$\begin{array}{c} x_{1j}\sim Binomial\left(3,p_{1j}\right)\\ x_{2j}\sim Binomial\left(3,p_{2j}\right)\\ x_{3j}\sim Binomial\left(3,p_{3j}\right)\\ p_{1j}=1-exp\left(-\lambda_{j}\times {df}_{1}\right)\\ p_{2j}=1-exp\left(-\lambda_{j}\times {df}_{2}\right)\\ p_{3j}=1-exp\left(-\lambda_{j}\times {df}_{3}\right)\\ C_{0}={log}_{10}\lambda_{j}\sim Normal\left(\mu_{C0},\sigma_{C0}\right) \end{array}$$

where *df*_1_, *df*_2_, and *df*_3_ were given the values of 0.1, 0.01, and 0.001, respectively. The prior for $\mu_{C_{0}}$ was defined as a non-informed Normal distribution with a mean of 1.0 and a standard deviation of $1/\sqrt{0.001}$; and the prior for $\sigma_{C_{0}}$ was set as a non-informed $1/\sqrt{Gamma\left(0.0001,0.0001\right)}$. Following a simulation of 20,000 iterations and a burn-in period of 5000 iterations, the mean value for $\mu_{C_{0}}$ was estimated at 1.038 log_10_ CFU/g (median: 1.058; standard deviation: 0.3061; 95% credibility interval: 0.379–1.590), and the mean value for $\sigma_{C_{0}}$ at 0.5436 log_10_ CFU/g (median: 0.4966; standard deviation: 0.3795; 95% credibility interval: 0.010–1.446).

**The R function:**

The function Lot2LotGen() generates a contamination matrix *N* of dimensions *r* × *c*, whose number of rows *r* represents the number of lots and can therefore be understood as a number of iterations that will correspond to between-lot variability and the number of columns *c* represents the number of units (frozen vegetable packs) of net weight *Unit_size_* grams to be produced in the lot, considered as fixed and equal for all lots. For every lot, *i* = {1, 2, …, *r*}, a value of prevalence *P_i_* is sampled from the between-lot prevalence distribution $Beta\;(\hat{\alpha },\;\hat{\beta })$. The number of contaminated units *s_i_* in a given lot *i* is determined as

$$s_{i}\;\sim \;Binomial\left(c,P_{i}\right)$$

and assigned to random locations, flagged with ones within the matrix *N*. The probability that the lot *i* is contaminated *Prob_UnitPos_* is estimated as

$${Prob}_{UnitPos\;i}=1-P\left(s_{i}=0\right)$$

considering that a lot is contaminated if at least one unit is contaminated. The mean prevalence of contaminated lots (*P*) is calculated as the complement of the beta-binomial probability evaluated at *s* = 0

$$P=1-\frac{\Gamma \left(c+\beta \right)}{\Gamma \left(c+\alpha +\beta \right)}$$

where $\Gamma \left(x\right)$ is the gamma function.

For the contaminated units, the numbers of *L. monocytogenes* are produced as follows. First, the mean concentration *C*_0*bi*_ for every lot *i* is sampled from,

$$C_{0bi}\;\sim \;Normal\left(\mu_{C0},\sqrt{{prop}_{Var}\times \sigma_{C0}^{2}}\right)$$

where *prop_Var_* is the proportion of the total variance *σ_C_*_0_^2^ attributed to between-lot variance (*prop_Var_* is assumed to be 0.7); and, secondly, the numbers of *L. monocytogenes* (*N_ij_*) in the contaminated units *s_i_* of the lot *i* is determined as

$$N_{ij}=\left\lceil {Unit}_{size}\times {10\;}^{max(Normal\left(C_{0bi},\sqrt{\left({1-prop}_{Var}\right)\times \sigma_{C0}^{2}}\right),\;\;\;{log}_{10}0.501/{Unit}_{size})}\right\rceil \;\;j=1,\;2\ldots ,\;s_{i}$$

The value of 0.501 is set to ensure that at least one bacterial cell is present in a contaminated unit. The non-contaminated units (*c*—*s_i_*) of every lot are assigned zero (CFU) in the matrix N. Apart from the parameters defining the between-lot prevalence (*α*, *β*) and concentration ($\mu_{C_{0}}$, $\sigma_{C_{0}}$) of *L. monocytogenes*, the function Lot2LotGen() requires other inputs, namely *nLots*, *sizeLot*, *Unit_size_*, and *prop_Var_*. The outputs of the function are the contamination matrix (*N*), the mean prevalence of contaminated lots (*P*, a scalar), and the probability that the iterated lot is contaminated (*Prob_UnitPo_*_s_, a vector).

#### 2.1.2. Blanching of Vegetables

**Data and assumptions:**

**Figure 1:**
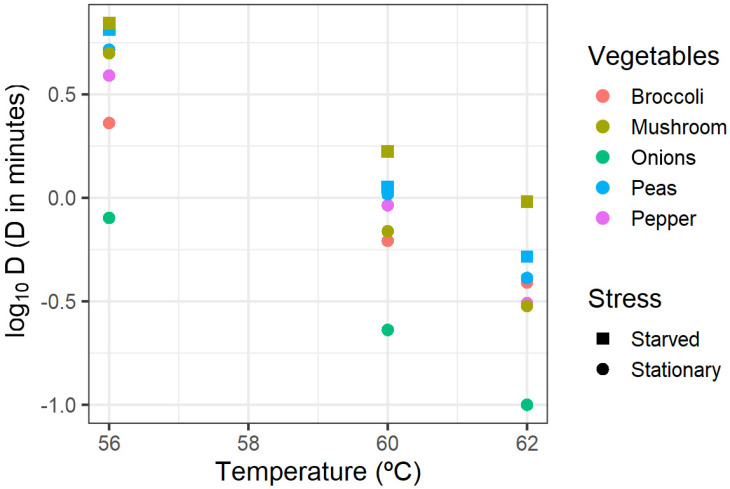
Heat resistance data of stationary-phase or starved *L. monocytogenes* inoculated in various vegetables, taken from Mazzotta [29], used to fit the Bigelow model for blanching.

Blanching is a technological heat treatment applied to vegetables before freezing, whose main objective is to inactivate enzymes that cause quality degradation. The level of reduction of *L. monocytogenes* during blanching is variable, as it depends on the vegetables and the process parameters applied (time/temperature); the latter are, however, neither regulated nor strictly defined [35]. Ceylan et al. [36] assessed the thermal inactivation of a cocktail of *L. monocytogenes* on peas, spinach, broccoli, potatoes, and carrots that were exposed to hot water and steam under specific time and temperature combinations. Although these authors presented many results, their reductions were all expressed in a “higher than x log” form, which made them unusable for the present QRA model. Data from Ceylan et al. (2017) could be used instead to compare the simulation outputs. Instead, the results from Mazzotta [29] were employed to model the inactivation kinetics of *L. monocytogenes* during blanching. Mazzotta [29] carried out challenge studies mimicking blanching to determine the heat resistance parameters of a cocktail of stationary-phase or starved *L. monocytogenes* cells (serotypes 1/2a, 1/2b, and 4b) in onions, broccoli florets, green peppers, mushrooms, and peas. The D values they determined at 56, 60, and 62 °C are shown in Figure 1.

The *L. monocytogenes* heat resistance data extracted from Mazzotta [29] in the context of evaluating the blanching process were modeled by adjusting a Bigelow equation of the form [37]

$$\log_{10}D=\log_{10}D_{ref}-\frac{\left(T-T_{ref}\right)}{z_{T}}$$

where *D* is the decimal reduction time in min, *T* is the treatment temperature (°C), *D_ref_* is the *D* (min) at the reference temperature (*T_ref_* = 70 °C), and *z_T_* is the z value (°C) or change in temperature required for a one-log_10_ reduction in *D*. Due to the inability to differentiate between stress effects and specific vegetable effects in the experimental setup, it was decided to incorporate all available data for the analysis. The parameter estimates were as follows: *log*_10_ *D_ref_* = −1.7840 (standard error = 0.2518) and *z_T_* = 6.061 (standard error = 0.7852), with a residual sum of squares 1.6815. For the QRA model, the variability in *log*_10_ *D_ref_* is defined as Normal (−1.784, 0.2518), and *z_T_* as a single point estimate (6.061). The mix of raw vegetables is assumed to be blanched at a temperature (*Temp_Blanch_*) of 83 °C for a duration (*time_Blanch_*) of 1.0 min.

**The R function:**

The function fvBlanching() simulates the effect of blanching on the numbers of *L. monocytogenes* present in the vegetables’ portions according to the Bigelow model explained above; and updates the probabilities of lots being contaminated (after blanching) and the mean prevalence of contaminated lots (after blanching). fvBlanching() is fed by the outputs of the function Lot2LotGen(), and by the blanching time and temperature (*time_Blanch_* [min], *Temp_Blanch_* [°C]). For every lot *i*, a value of *log*_10_ *D_ref i_* is sampled from the normal distribution, and the probability of survival of a microbial cell *p_SurviveBlanching i_* for that lot is determined as

$$p_{SurviveBlanchingi}=10^{{time}_{Blanch}\times \left(\log_{10}D_{refi}-\frac{\left({Temp}_{Blanch}-70\right)}{6.061}\right)}$$

Understanding *p_SurviveBlanching i_* as a binomial probability, the number of surviving cells after blanching *N_blanched ij_* can be estimated as,

$$N_{Blanched\;ij}\;\sim \;Binomial\left(N_{ij},p_{SurviveBlanching\;i}\;\right)\;\;\;\;\;\;\;\;j=1,\;2\ldots ,\;c$$

It is noteworthy mentioning that $p_{SurviveBlanching\;}$ does not include the come-up time in its calculation. To update the prevalence estimates after blanching, the probability that at least one cell survives in a lot (*atLeastOne_i_*) is calculated:

$${atLeastOne}_{i}=1-{\left(1-p_{SurviveBlanching\;i}\right)}^{\sum_{j=1}^{c}N_{ij}}$$

The probability vector of contaminated lots after blanching *Prob_UnitPos blanched i_* is then updated,

$${Prob}_{UnitPosblanchedi}={Prob}_{UnitPosi}\times {atLeastOne}_{i}$$

Likewise, the mean prevalence of contaminated lots P_Blanched_.

$$P_{Blanched}=P\times \frac{\sum_{i=1}^{r}{atLeastOne}_{i}}{r}$$

The outputs of the function fvBlanching() are the contamination matrix (numbers of *L. monocytogenes* per unit) after blanching (*N_Blanched_*), the mean prevalence of contaminated lots after blanching (*P_Blanched_*, a scalar), and the probabilities that the sampled lots are contaminated (*Prob_UnitPos blanched_*, a vector).

#### 2.1.3. Freezing and Packaging

**Data and assumptions:**

Studies have shown that there is a probability for cross-contamination to occur after blanching from equipment in contact with food, such as post-blanching conveyor belts, freezing tunnels, and packaging machines [3,38]. Furthermore, non-food contact surfaces and harboring sites play a role in the spread of *L. monocytogenes* to the final product [3,22,39]. Since freezing tunnels and packaging machines have been associated with high recoveries of *L. monocytogenes* in processing facilities (12.5% in Truchado et al. [3]; 36.4% in Magdovitz et al. [22]; 41.3% in Pappelbaum et al. [38]), the present QRA model was designed to accommodate the possibility of bacterial transfer from surfaces such as conveyor belts, freezing tunnels and/or packaging machines. Thus, for the model, the overall risk of post-blanching recontamination is considered as one process. The probability of bacterial transfer occurring after blanching, *P_cc_*, was set to the value provided by the most recent source, Truchado et al. [3] (0.125). The distribution for the transfer coefficient of *L. monocytogenes* from stainless steel to vegetables [Normal (−0.44, 0.40) in log_10_] reported by Hoelzer et al. [30] was employed in the QRA model. Following the potential recontamination event, the frozen bulk vegetables are packaged into bag units, which, in modeling terms, involve the representation of the microbial process of partitioning. In a contaminated lot, *L. monocytogenes* is assumed to be moderately clustered in the bulk of frozen vegetables; therefore, a dispersion factor *b* = 1 is assumed [31].

**The R function:**

The function fvPartitioningCC() simulates two microbial processes: firstly, microbial transfer by the potential recontamination of blanched vegetables when in direct contact with contaminated surfaces between transport by conveyors and packaging and subsequently, partitioning of the bulk of frozen vegetables into packed units. fvPartitioningCC() is fed by the outputs of the function fvBlanching(), and by the probability of recontamination (*P_cc_*), the dispersion factor (*b_CCFV_*), the parameters of the normal distribution about the log_10_ transfer rate from stainless steel to vegetables (*TR_equip-veg_*), and the load of *L. monocytogenes* cells on the surface of conveyors, freezer and/or packaging machine ready to be transferred (*N_equip_*).

The algorithm starts by calculating the total numbers of *L. monocytogenes* in every lot *i* before the potential recontamination event (*N_tot_*),

$$N_{tot\;i}=\sum_{j=1}^{c}N_{ij}$$

If the recontamination event takes place, at a probability *P_cc_*, a fraction (*TR_equip-veg_*) of the numbers of cells on the contaminated equipment surface(s) (*N_equip_*) is transferred to the bulk of vegetables (*N_from-equip_*). The algorithm considers that if the bulk of vegetables is not contaminated with *L. monocytogenes* (actually not represented in the contamination matrix), it may become contaminated from equipment; and if the lot is contaminated, its load will increase or remain the same depending on the recontamination event happening or not, respectively. This raises three possible scenarios for contaminated lots [40]: (1) no recontamination occurring in lots already contaminated (*Bulk_Pos__CC_Neg_*); (2) recontamination occurring in lots already contaminated (*Bulk_Pos__CC_Pos_*); and (3) recontamination occurring in lots that were not contaminated (*Bulk_Neg__CC_Pos_*). The fourth case, represented by the status of the non-contaminated lot (*Bulk_Neg__CC_Neg_*), is not taken into account in the upcoming calculations since it does not affect the microbial numbers.

The probability of each of the three contaminated lots taking place is calculated as

$$P\left({Bulk}_{Pos}\_{CC}_{Neg\;i}\right)={Prob}_{UnitPosblanchedi}\times \left(1-P_{cc}\right)$$

$$P\left({Bulk}_{Pos}\_{CC}_{Pos\;i}\right)={Prob}_{UnitPosblanchedi}\times P_{cc}$$

$$P\left({Bulk}_{Neg}\_{CC}_{Pos\;i}\right)=\left(1-{Prob}_{UnitPosblanchedi}\right)\times P_{cc}$$

The status of every lot (*i* = 1, 2,…, *c*) is then randomly sampled from the probabilities {$P\left({Bulk}_{Pos}\_{CC}_{Neg\;i}\right),\;P\left({Bulk}_{Pos}\_{CC}_{Pos\;i}\right),\;P\left({Bulk}_{Neg}\_{CC}_{Pos\;i}\right)$}. This strategy is adopted to maintain the dimension [*r*, *c*] of the input contamination matrix $N_{Blanched\;}$. The extent of recontamination in CFU per lot *i* is computed as

$${TR}_{equip-veg\;i}\;\sim \;Normal\;\left(-0.44,\;0.40\right),\;\;\;\;{TR}_{i}\leq 0$$

$$N_{from-equip\;i}\;\sim \;Binomial\left(N_{equip},\;10^{{TR}_{equip-veg\;i}}\right),\;\;\;\;N_{from-equip\;i}>0$$

where *N_equip_* can be understood as the total load of *L. monocytogenes* cells on the surface of conveyors, freezer and/or packaging machine in contact with the bulk of vegetables. Next, depending on the iterated lot status, the value of *N_from-equip i_* is added to *N_tot i_* (or not), in order to determine the numbers of *L. monocytogenes* in the lot *i* after the potential recontamination event (*N_tot postCC i_*), according to

$$N_{tot\;postCC\;i}=\left\{\begin{array}{cc} N_{tot\;i} & i\in {Bulk}_{Pos}\_{CC}_{Neg}\\ N_{tot\;i}+N_{from-equip\;i} & i\;\in \;{Bulk}_{Pos}\_{CC}_{Pos}\\ N_{from-equip\;i} & i\in {Bulk}_{Neg}\_{CC}_{Pos} \end{array}\right.$$

Next, the partitioning algorithm randomly distributes the total numbers of cells *N_tot postCC i_* from a contaminated lot into *c* packed units, following a Dirichlet-multinomial process:

$${\left[\begin{array}{ccc} p_{dist\;1}\;\;\;p_{dist\;2} & \ldots & p_{dist\;c} \end{array}\right]}_{i}\;\sim \;Dirichlet\left({\left[\begin{array}{ccc} b_{j=1}\;\;\;\;\;b_{j=2}\; & \ldots & b_{j=c} \end{array}\right]}_{i}\right)\;\;\;\;i=1,\;2,\;\ldots ,\;r$$

$${\left[N_{pack\;1}\;\;\;N_{pack\;2}\;\;\;\cdots \;\;\;N_{pack\;c}\right]}_{i}\;\sim \;Multinomial\left(N_{tot\;postCC\;i},\;{\left[p_{dist\;1}\;\;\;p_{dist\;2}\;\;\;\cdots \;\;\;p_{dist\;c}\right]}_{i}\right)\;\;\;\;\;\;\;\;\;\;\;\;\;\;\;\;\;\;\;\;\;\;\;\;\;\;\;\;\;i=1,\;2,\;\ldots ,\;r$$

A fixed value for the parameter b is used in order to avoid increasing the uncertainty in the model’s outputs, given the lack of data about the dispersion of *L. monocytogenes* in the bulk of frozen vegetables.

The resulting matrix *N_pack_* contains the numbers of *L. monocytogenes* cells in *c* packs of frozen vegetables (horizontal dimension) in a sample of *r* contaminated lots (vertical dimension). Finally, the probability vector of contaminated lots after packaging *Prob_UnitPos pack i_* and the mean prevalence of contaminated lots after packaging *P_Pack_* are updated.

$${Prob}_{UnitPospacki}=1-\left(1-{Prob}_{UnitPos\;blanchedi}\right)\times \left(1-P_{cc}\right)$$

$$P_{Pack}=1-\left(1-P_{Blanched}\right)\times \left(1-P_{cc}\right)$$

The outputs of the function fvPartitioningCC() are the matrix *N_Pack_*, the vector *Prob_UnitPos pack_* and the scalar *P_Pack_*.

#### 2.1.4. Post-Packaging Treatment

**Data and assumptions:**

Although the application of a post-packaging inactivation treatment for frozen foods is challenging [41], a generic post-processing inactivation treatment was considered to represent any alternative approach to reduce *L. monocytogenes* risk during processing [42]. The log_10_ reduction attained by the generic treatment is assumed to follow a triangular distribution with a minimum value of *Interv_min_*, a mode of *Interv_mode_*, and a maximum value of *Interv_max_* representing the variability in *L. monocytogenes* inactivation. In the current model, such values were set to zero to assume there is no post-packaging treatment.

**The R function:**

The fvReductionPostpack() function receives the outputs of the function fvPartitioningCC() and the values of *Interv_min_*, *Interv_mode_*, and *Interv_max_* set by the user. For every lot *i*, the probability of survival of a microbial cell *p_SurvivePostpack i_* is sampled as

$$p_{SurvivePostpack\;i}=10^{-Triang\;\left({Interv}_{min},\;{Interv}_{mode},{Interv}_{max}\right)}$$

and the number of surviving cells after the inactivation treatment *N_Interv_* is obtained, under a binomial process assumption, by

$$N_{Interv\;ij}\;\sim \;Binomial\left(N_{pack\;ij},p_{SurvivePostpack\;i}\;\right)\;\;\;\;\;\;\;\;j=1,\;2\ldots ,\;c$$

To update the prevalence estimates after the inactivation treatment, the probability of at least one cell surviving in the lot *i* (*atLeastOne_i_*) is calculated:

$${atLeastOne}_{i}=1-{\left(1-p_{SurvivePostpack\;i}\right)}^{\sum_{j=1}^{c}N_{ij}}$$

The probability vector of contaminated lots after the inactivation treatment *Prob_UnitPos interv i_* and the mean prevalence of contaminated lots *P_Interv_* are then updated:

$${Prob}_{UnitPosintervi}={Prob}_{UnitPos\;packi}\times {atLeastOne}_{i}$$

$$P_{Interv}=P_{Pack}\times \frac{\sum_{i=1}^{r}{atLeastOne}_{i}}{r}$$

#### 2.1.5. Within-Lot Testing

**Data and assumptions:**

The QRA model enables the microbiological testing of food unit samples taken from a lot, according to a two-class or a three-class mixed sampling plan [43]. In a two-class plan (the most commonly used for *L. monocytogenes*), *n* units are randomly sampled and analyzed per lot. For each sampled unit, a quantity of *g* grams is used in the enrichment essay and tested. The lot is rejected if more than *c* sampled units are positive in detection. In a three-class mixed sampling plan, sampled units are also enumerated, and the lot is rejected if more than *c* sampled units are positive in detection or if at least one sampled unit has an estimated concentration greater than a predefined concentration *M* [44]. It is assumed that the enumeration test is conducted only on positive samples in detection by direct plating of $g_{TestedEnum}$ g taken from the same sample.

**The R function:**

The function fvTesting() receives the outputs of the function fvReductionPostpack(), *n*, the number of tested units, *g* the sub-sample weight in grams used for detection, *c* the number of positive samples accepted (two-class or three-class mixed plan), *M* the maximum limit concentration, *p_lot tested_*, the proportion of tested lots, *Se*, the probability of the test (enumeration or detection) to detect, independently, each bacteria present in a sample, and *g_TestedEnum_*, the sub-sample weight in grams used for the enumeration essay.

Assuming perfect homogenization of the sample, each of the bacteria present in each of the *r* × *c* units of *Unit_size_* weight has a probability of being present in the *g* grams of the sub-sample and detected equal to $Se\times {g/Unit}_{size}$. The number of bacteria detected in the detection essay is then

$$N_{detected\;ij}\;\sim \;Binomial\left(N_{Pack\;ij},Se\times {g/Unit}_{size}\right)$$

and the detection test is positive if $N_{detected\;ij}>0$.

If the sampling plan is a 3-class plan, an enumeration test (direct plating) is performed. The number of bacteria enumerated in the sample is

$$N_{enumerated\;\;ij}\;\sim \;Binomial\left(N_{Pack\;ij},Se\times {g_{TestedEnum}/Unit}_{size}\;\right)$$

and the estimated concentration is $N_{enumerated\;ij}/g_{TestedEnum}$ CFU/g. The algorithm assumes that the enumeration is performed only on samples positive in detection.

In order to evaluate the probability of each of the lot to be rejected, 1000 (by default) Monte Carlo samples of *n* samples are constituted for each lot and, for each of these Monte Carlo samples, the microbiological criteria is applied (i.e., in a 2-class plan, the test is positive if >*c* samples among *n* are detected; whereas in a 3-class plan, the test is positive if >*c* samples among *n* are detected or if at least one sample has an estimated concentration >*M* CFU/g). The mean number of times the lot is rejected among the Monte Carlo samples multiplied by the probability for the lot to be tested is an estimate of *P_pos i_*, the probability of the lot *i* to be rejected.

The function updates the prevalence outputs using the Bayes’ theorem. Given the probability of the lot *i* being contaminated pre-testing (*Prob_UnitPos interv i_*) and the probability for the lot *i* to be rejected (*P_pos i_*), the prevalence of contaminated lots given that they were not detected after testing (*Prob_UnitPos tested i_*) is

$${Prob}_{UnitPos\;tested\;i}=\frac{\left(1-P_{pos\;i}\right)\times {Prob}_{UnitPos\;interv\;i}}{\left(1-P_{pos\;i}\right)\times {Prob}_{UnitPos\;interv\;i}+\left(1-{Prob}_{UnitPos\;interv\;i}\right)}$$

The mean prevalence of contaminated lots after within-lot testing (*P_Tested_*, a scalar) is therefore,

$$P_{Tested}=\frac{\left(1-\frac{\sum_{i=1}^{r}P_{pos\;i}}{r}\right)\times P_{Interv}}{\left(1-\frac{\sum_{i=1}^{r}P_{pos\;i}}{r}\right)\times P_{Interv}+\left(1-P_{Interv}\right)}$$

Contaminated lots detected after testing are not removed from the matrix, and therefore the input matrix *N_Interv_* is returned unchanged. The function fvTesting() only updates the prevalence outputs.

#### 2.1.6. Portioning

**Data and assumptions:**

The product is expected to be kept frozen during distribution and retail, and therefore, no growth of *L. monocytogenes* in these stages is modeled. During handling at home, it is assumed that the consumer removes a portion *Serv_size_* (g) from the pack of frozen vegetables of net weight *Unit_size_* g. Such a portion later becomes the serving size. The portion *Serv_size_* is assumed to be 50 g, as in the model of EFSA [7]. Then, the number of *L. monocytogenes* cells in this small unit can be considered to be a sample from a Beta-binomial distribution because only one portion per pack is retained (as opposed to simulating all portions that can be obtained from a pack). A moderate clustering of *L. monocytogenes* cells in the frozen vegetables contained in the pack is assumed (dispersion *b* = 1 [31]). The dispersion parameter and the number of portions that can be obtained from a pack are assumed to be independent of the microbial numbers. The likelihood that a non-contaminated portion of frozen vegetables can be obtained from a contaminated pack of frozen vegetables is taken into account.

**The R function:**

The function fvPortioning() mimics the microbial process of partitioning; yet, differently from the function fvPartitioningCC(). The numbers of *L. monocytogenes* in a portion (*N_Portion ij_*) taken from the pack *j* produced in the lot *i* is sampled from a beta-binomial distribution

$$N_{Portion\;ij}\;\sim \;Binomial\left(N_{Interv\;ij},\;Beta\left(b,b\left(n_{serv}-1\right)\;\right)\right)$$

where *n_serv_* is the rounded number of servings from a pack of frozen vegetables,

$$n_{serv}=\left\lceil \left.\frac{{Unit}_{size}}{{Serv}_{size}}\right\rceil \right.$$

Next, the probability that a contaminated pack *ij* produces a non-contaminated portion (*π*_0 __*ij*_) is calculated as the beta-binomial probability of *N_portion ij_* = 0.

$$\pi_{0\;ij}=\frac{\Gamma (b\times n_{serv})\times \Gamma \left(N_{Packij}+b\times \left(n_{serv}-1\right)\right)}{\Gamma \left(b\times \left(n_{serv}-1\right)\right)\times \Gamma (b\times n_{serv}+N_{Packij})}$$

The mean probability of non-contaminated portions from a lot *i* (${\bar{\pi }}_{0\;i}$) is computed as,

$${\bar{\pi }}_{0\;i}=\frac{\sum_{j=1}^{c}\pi_{0\;ij}}{c}$$

and is used to estimate the vector of lot-specific probabilities of contaminated portions *Prob_UnitPos portion_* and the overall prevalence of contaminated portions (*P_Portion_*, a scalar).

$${Prob}_{UnitPosportioni}={Prob}_{UnitPos\;testedi}\times {\bar{\pi }}_{0\;i}$$

$$P_{Portion}=P_{Tested}\times \frac{\sum_{i=1}^{r}{\bar{\pi }}_{0i}}{r}$$

Such probabilities and the contamination matrix of frozen vegetable portions (*N_Portion_*) are the outputs of the function fvPortioning(). The size of the contamination matrix remains as [*r*, *c*].

#### 2.1.7. Defrosting

**Data and assumptions:**

*L. monocytogenes* can survive for long periods of time in frozen foods [38] and resume growth when defrosted [35]. To this respect, Kataoka et al. [45] have shown that the lag phase time required for *L. monocytogenes* to start growth depends on the defrost/storage temperature and the type of vegetable. For instance, these authors found that, at ambient temperature (20 °C), the lag phase duration of a cocktail of *L. monocytogenes* (1/2a, 1/2b, 1/2c, 4b) inoculated in corn (0.53 h) was significantly lower than that of peas (3.40 h). On the other hand, EFSA [7] has indicated that the blanching treatment would lead to a product more supportive of *L. monocytogenes* growth (if re-contaminated) during subsequent storage after thawing. This is likely due to the absence of an antagonistic effect by the background microflora, which have been inactivated during heat treatment.

The present QRA model disregards the occurrence of a lag phase and assumes a log-linear growth of *L. monocytogenes* in the defrosted portion taken from the pack of frozen vegetables. The lognormal distribution of the exponential growth rate of *L. monocytogenes* in heat-treated vegetables at a reference temperature of 5 °C (*EGR*_5*C*_; log_10_/h) was taken from EFSA [7], which was built using growth data collected from the scientific literature on *L. monocytogenes* in blanched vegetables including corn, green peas, carrots, broccoli, beans and asparagus stored in air, later converted to EGR_5C_ using the square-root secondary model [46]. Thus, it is considered that such a distribution involves the variability between vegetables, between *L. monocytogenes* strains, and between effects of blanching treatments. The mean *μ_EGR_*_5*C*_ = 0.0117 and standard deviation *σ_EGR_*_5*C*_ = 0.00816 in log_10_/h of the lognormal distribution for EGR_5C_, proposed in EFSA [7], were converted to the log_e_ scale for use as parameters of the lognormal distribution (−4.646, 0.6296, respectively). Whereas the *L. monocytogenes* minimum temperature for growth (*T_min_*) was regarded as −1.18 °C [7,46], the maximum population density (MPD) in thawed vegetables was assumed to be 8.0 log_10_ CFU/g [45].

There is limited information on the consumers’ way of preparing non-RTE frozen vegetables, except for the surveys conducted in the UK [33] and later in Ireland [34]. In Ireland, the survey participants were asked about their preparation habits, and a high proportion (84–89%) recognized that vegetables such as sweet corn, peas, mushrooms, and mixed vegetables needed to be cooked prior to consumption. Those who consumed non-RTE products without cooking (~20%) indicated that their common ways of preparation were in salads and as garnish [34]. In this way, the present QRA model considered that the probability of defrosting the portion of vegetables (*p_Defrost_*) is 0.20 since it can be assumed that 80% of the time, the consumers cook the vegetables directly from the frozen state. If defrosted, the user-selected time-temperature (*Time_Defrost_* [h], *Temp_Defrost_* [°C]) combinations for defrosting/storage may reflect several consumer practices such as placing the frozen vegetables on the counter-top at room temperature, in the refrigerator for overnight defrost, or using the microwave for quick defrost. It will be assumed that consumers leave frozen vegetables to defrost for a couple of hours on the countertop 20% of the time (*Temp_Defrost_* = 25 °C, *Time_Defrost_* = 2 h, and *p_Defrost_* = 0.20).

**The R function:**

The fvDefrost() function simulates the growth of *L. monocytogenes* in the vegetables during defrosting and is fed by the outputs of the function fvPortioning() along with *Time_Defrost_*, *Temp_Defrost_*, *p_Defrost_*, and *Unit_size_*. The algorithm begins by randomly allocating the portions of frozen vegetables that will be defrosted by the consumer at a probability *p_Defrost_*, which is independent of the lot. According to a Bernoulli distribution with parameter *p_Defrost_*, the defrost status matrix *Defrost* is created, where *Defrost_ij_* = 1 represents the defrosted portions and *Defrost_ij_* = 0 the non-defrosted ones.

$${Defrost}_{ij}\;\sim \;Bernoulli\left(p_{Defrost}\right)\;\;\;\;\;\;\;i=1,\;2\ldots ,\;r;\;\;j=1,\;2\ldots ,\;c$$

For the portions *Defrost_ij_* = 1, the *L. monocytogenes* growth in log_10_ CFU/g is estimated as,

$${Delta}_{ij}\;\sim \;\;lognormal\left(\mu_{EGR5C},\sigma_{EGR5C}\right){\left(\frac{{Temp}_{Defrost}-T_{min}}{5-T_{min}}\right)}^{2}\times {Time}_{Defrost}$$

and the matrix *N_Defrost_* of the numbers of *L. monocytogenes* after thawing is finally computed taking into account the MPD restriction:

$$N_{Defrost\;ij}=\left\{\begin{array}{c} {Unit}_{size}\times 10^{\left(\log_{10}\frac{N_{Portion\;ij}}{{Unit}_{size}}+{Delta}_{ij}\right)},\;\;if\;{Unit}_{size}\times 10^{\left(\log_{10}\frac{N_{Portion\;ij}}{{Unit}_{size}}+{Delta}_{ij}\right)}<{Unit}_{size}\times 10^{MPD}\\ {Unit}_{size}\times 10^{MPD},\;\;if\;{Unit}_{size}\times 10^{\left(\log_{10}\frac{N_{Portion\;ij}}{{Unit}_{size}}+{Delta}_{ij}\right)}\geq {Unit}_{size}\times 10^{MPD} \end{array}\right.$$

The function acts only on the contaminated portions (*N_Portion ij_* > 0), producing the contamination matrix *N_Defrost_*, and returning unaffected the vector of lot-specific probabilities of contaminated portions (*Prob_UnitPos portion_*) and the overall prevalence of contaminated portions (*P_Portion_*, a scalar). It is noteworthy to mention that the growth model does not allow for the come-up time.

#### 2.1.8. Cooking

**Data and assumptions:**

According to a survey at retail in the UK [33], 77% of the frozen vegetable packages recommended cooking before consumption, 4% specified being RTE, and on 19% of the packages, there was no indication for cooking. In Ireland, 9.3% of the frozen vegetable samples did not have any non-RTE label or cooking instructions printed on the packaging indicating that frozen vegetables must be fully cooked. Of public health impact concern is tackling those non-intended uses of non-RTE frozen vegetables as alternatives of consumption. The present QRA model assumed in its scenarios a probability of cooking a serving (*p_Cook_*) of 1 (always cooked) or 0 (never cooked).

It is expected that the extent of inactivation of *L. monocytogenes* depends on the time of cooking and type of cooking. In EFSA’s generic model [7], the reduction by cooking was assumed to follow a Pert distribution with a minimum 1.0 log_10_, mode 3.0 log_10_, and maximum 9.0 log_10_. The present QRA model represented such variability as a triangular distribution (*Cook_min_*, *Cook_mode_*, *Cook_max_*) with the same parameters.

**The R function:**

The function fvCooking() simulates the microbial process of reduction of *L. monocytogenes* in the (defrosted) vegetables during cooking. It takes the outputs of the function fvDefrost() and the parameters *p_Cook_*, *Cook_min_*, *Cook_mode_*, *Cook_max_*. The algorithm starts by randomly flagging the portions of defrosted vegetables that will be cooked, regardless of the lot or their contamination status. According to Bernoulli distribution with parameter *p_Cook_*, the cooked status matrix *Cook* is produced, where *Cook_ij_* = 1 represents the portions to be cooked, and *Cook_ij_* = 0 the ones that will not.

$${Cook}_{ij}\;\sim \;Bernoulli\left(p_{Cook}\right)\;\;\;\;\;\;\;i=1,\;2\ldots ,\;r;\;\;j=1,\;2\ldots ,\;c$$

For the portions *Cook_ij_* = 1, the probability of survival of a microbial cell *p_SurviveCooking ij_* is sampled as,

$$p_{SurviveCooking\;ij}=10^{-Triang\;\left({Cook}_{min},\;{Cook}_{mode},{Cook}_{max}\right)}$$

and the number of surviving cells after cooking *N_Cook_* is obtained by,

$$N_{Cook\;ij}\;\sim \;Binomial\left(N_{Defrost\;ij},p_{SurviveCooking\;ij}\;\right)\;\;\;\;\;\;\;N_{Cook\;ij}>0\;$$

To update the prevalence estimates after cooking, the probability of at least one cell surviving in a cooked portion (*atLeastOne_ij_*) is calculated:

$${atLeastOne}_{ij}=1-{\left(1-p_{SurviveCooking\;ij}\right)}^{N_{Defrost\;ij}}$$

The probability vector of contaminated lots after cooking *Prob_UnitPos cooked i_* and the mean prevalence of contaminated portions (after the potential cooking event) *P_Cooked_* are then updated,

$${Prob}_{UnitPoscookedi}={Prob}_{UnitPos\;portioni}\times \left\{\left(1-p_{Cook}\right)+p_{Cook}\times \frac{\sum_{i=1}^{c}{atLeastOne}_{ij}}{c}\right\}$$

$$P_{Cooked}=P_{Portion}\times \left\{\left(1-p_{Cook}\right)+p_{Cook}\times \frac{\sum_{i=1}^{r}\sum_{j=1}^{c}{atLeastOne}_{ij}}{r\times c}\right\}$$

The function fvCooking() returns the contamination matrix *N_Cooked_* containing the load of *L. monocytogenes* in the servings originated from the contaminated lots, the lot-specific probability of contaminated servings to be consumed (*Prob_UnitPos cooked_*, a vector) and the mean prevalence of contaminated servings to be consumed (*P_Cooked_*, a scalar).

### 2.2. Risk Characterization

Several dose-response relationships are available for *L. monocytogenes* [47]. For purposes of comparison with the model of Zoellner et al. [8], the dose-response model chosen to estimate the risk of listeriosis per serving in the susceptible population was that of FAO-WHO [48]. According to this exponential model, each ingested *L. monocytogenes* cell has an independent probability *r* of causing invasive listeriosis, which is assumed constant within a given population. FAO-WHO [48] inferred a median value for *r* of 1.06 × 10^−12^ for the “population with increased susceptibility”. The function DRQuick() from the doseresponsemodels R package [47] was employed to calculate the marginal probabilities of invasive listeriosis in the susceptible population *RiskServing_ij_* from the input matrix *N_Cooked ij_*, containing the *L. monocytogenes* doses (CFU) in servings *j* from contaminated lots *i*. The mean risk for every lot *i* (*RiskLot_i_*) was calculated as a risk averaged across servings *j*, and weighed by the lot-specific probability *Prob_UnitPos cooked i_*:

$${RiskLot}_{i}=\frac{\sum_{j=1}^{c}{RiskServing}_{ij}\times {Prob}_{UnitPos\;cooked\;i}}{c}$$

### 2.3. QRA Model’s Ouputs

The model’s outputs were summarised at three stages: end of processing, end of exposure assessment (point of consumption), and risk characterization. The descriptors at the *end of processing* were calculated from the outputs of the function fvTesting(), and included descriptive statistics (mean, median, and 95% confidence interval) of the mean concentration of *L. monocytogenes* (CFU/g) in the fraction of contaminated lots, the prevalence of contaminated packs, the probability that a contaminated pack contains more than 10 CFU *L. monocytogenes* per g frozen vegetables, and the probability that a contaminated pack contains more than 100 CFU *L. monocytogenes* per g frozen vegetables. The model’s descriptors at the *point of consumption* were computed from the outputs of the function fvCooking(), and encompassed descriptive statistics (mean, median, and 95% confidence interval) of the concentration of *L. monocytogenes* in any serving, the prevalence of contaminated servings, the probability that a contaminated serving contains more than 10 CFU *L. monocytogenes* per g vegetables, and the probability that a contaminated serving contains more than 100 CFU *L. monocytogenes* per g vegetables. The descriptors for risk characterization include the summary statistics, mean, median, and 2.5 and 97.5 percentiles of the lot-level mean risk per serving *RiskLot*.

### 2.4. QRA Model’s Functionality: Reference and What-If Scenarios

To illustrate the utility of the QRA model, a reference scenario was run, in addition to three processing-related scenarios, which simulated changes in the initial *L. monocytogenes* contamination of the vegetables, in the blanching temperature, and in the contamination level of the processing environment.

(a) Reference, which is the baseline scenario constituted of parameters whose values represent as much as possible the current situation supported by actual data and, in their absence, reasonable assumptions.
(b) Initial contamination, assessed by scenarios representing mean initial *L. monocytogenes* concentrations in pre-blanched vegetables that are lower (*μ_C_*_0_ − 1 log_10_ CFU/g) or higher (*μ_C_*_0_ + 1 log_10_ CFU/g) than the reference scenario (*μ_C_*_0_ = 1.038 log_10_ CFU/g).
(c) Blanching temperature, assessed by scenarios with temperatures lower than the reference scenario (*Temp_Blanch_* = 71 and 77 °C).
(d) Recontamination after blanching, evaluated by scenarios representing total loads of *L. monocytogenes* on equipment surfaces after blanching in a lot, lower (*N_equip_*/100) and higher (N_equip_*100) than the reference scenario (*N_equip_* = 10000 CFU).

To assess the impact of the most important consumer’s handling practice (i.e., cooking the non-RTE product), all of the above scenarios were run in two modalities, assuming that vegetables are not cooked (*p_Cook_* = 0) and that vegetables are always cooked (*p_Cook_* = 1). Models were run for 2000 lots (*r*) and 5000 units per lot (*c*).

### 2.5. QRA Model’s Implementation

All the functions described in Section 2.1 were programmed in R version 4.4.1 [49] and compiled in the package qraLm, which can be installed from the Github repository: https://github.com/WorldHealthOrganization/qraLm, accessed on 10 September 2024. The reference manual can be found at: https://WorldHealthOrganization.github.io/qraLm/reference/, accessed on 10 September 2024.

## 3. Results and Discussion

### 3.1. Reference Scenario and Comparison with Other QRA Models

According to the reference scenario, the mean lot concentration of *L. monocytogenes*, determined after simulating 2000 lots, was found to be very low (95% credibility interval: 8.0 × 10^−7^–2.5 × 10^−4^) with an overall prevalence of 9.40% of contaminated 500 g packs at the end of processing. Furthermore, virtually all of these contaminated packs would have *L. monocytogenes* concentrations lower than 10 CFU/g (Table 3). The mean concentration in a contaminated pack at the end of processing would be 3.35 × 10^−4^ CFU/g (95% credibility interval: 0–0.004 CFU/g).

Surveys’ data on the prevalence of *L. monocytogenes* in non-RTE frozen vegetables sampled at the end of processing and retail displayed variable estimates (Table 4), ranging from 0% [25,38,50,51,52] to 45.6% [38], yet a random-effects meta-analysis of the ten primary sources (Table 4) produced a pooled estimate (5.66%; 95% CI: 2.28–13.3%) that was not distant from the prevalence estimated by the QRA model (9.40%). In terms of enumeration analysis, Moravkova et al. [53], Willis et al. [33], and FSAI [34] found that all *L. monocytogenes*-positive samples presented concentrations below 100 CFU/g, which is also in agreement with the reference scenario of the QRA model.

The simulation for the reference scenario predicts that the *L. monocytogenes* prevalence in uncooked 50 g servings is 1.37%, although such contaminated servings would have a very low probability of exceeding the concentration of 10 CFU/g (0.0046%; Table 5). In comparison with the QRA model of Zoellner et al. [8], our model estimated at least a five-fold lower number of *L. monocytogenes* in a contaminated serving, whereas Zoellner et al. [8] predicted a median of 32 CFU in a 140 g uncooked serving, our model estimated a median of 2 CFU (95% CI: 1.0–6.0 CFU) in a 50 g uncooked contaminated serving (results not tabulated).

According to our model, cooking the non-RTE vegetables decreases the prevalence of *L. monocytogenes* in 50 g servings to 0.0026%, yielding for the mean count in any serving a negligible value of 5.21 × 10^−7^ CFU/g (Table 5). In our model, if all servings were uncooked, the mean risk of listeriosis per serving in the susceptible population would be 2.93 × 10^−14^ (median 3.50 × 10^−15^; 95% CI: 1.28 × 10^−17^–1.61 × 10^−13^); which reveals a risk level far lower than the one predicted by the model of Zoellner et al. [8] for uncooked servings (median 7.8 × 10^−12^ in the susceptible population).

The higher levels of risk obtained in the model of Zoellner et al. [8] can be explained at least by three facts: (1) the higher probability of thawing (0.5, as opposed to 0.2 in our model); (2) the possibility of longer storage at room temperature (from 0.25 to 10 h, as opposed to a fixed value of 2 h in our model); and (3) the absence of the processing stage of blanching in their model, which is a step that largely reduces the initial contamination.

To this respect, in our model, blanching decreased the mean *L. monocytogenes* load in contaminated lots from 1.417 CFU/g in pre-blanched vegetables to 0.00020 CFU/g in post-blanch vegetables. In turn, the QRA model of Zoellner et al. [8] begins from the *L. monocytogenes* concentration in the lot after manufacturing (post-blanch vegetables), assuming a mean of 0.30 CFU/g, which is 1500-fold higher than our post-blanching mean concentration. Moreover, through sensitivity analysis, these authors concluded that it was the high initial *L. monocytogenes* concentration in the manufactured lot that was the most important factor driving the high risk of listeriosis in the uncooked servings.

In the present QRA model, cooking the non-RTE frozen vegetables decreases the mean risk of listeriosis per serving in a lot by 3.0 log_10_, from 2.935 × 10^−14^ (uncooked servings) to 2.765 × 10^−17^ (cooked servings; Table 6); which is an outcome in line with the findings of Zoellner et al. model [8], who found risk levels lower than 10^−16^ linked to the cooked servings (actual mean risk value not presented by these authors given the floating point limitation of the simulation software used).

The model of EFSA [7] also assessed consumer’s handling scenarios, and in the case of cooking, they predicted that in contrast to consuming uncooked servings, the consumption of cooked servings reduces the mean risk of listeriosis by 3.2 log_10_ in the female susceptible population and by 3.5 log_10_ in the male susceptible population, which are both reduction levels of comparable magnitude to our estimation. Nonetheless, in absolute terms, EFSA’s model [7] predicted higher mean risk of illness at 4.0 × 10^−10^ and 1.9 × 10^−9^ for uncooked servings and 2.3 × 10^−13^ and 5.3 × 10^−13^ for cooked servings, in the female and male susceptible populations, respectively.

The higher level of listeriosis risk per serving estimated by EFSA [7]—in contrast with our results—arises as a consequence of their more conservative assumptions, namely (1) a higher initial mean concentration (which can reach up to 5 log_10_ CFU/g); (2) the longer time between vegetables being defrosted and consumed for the uncooked servings’ risk estimation (mean of 9 h); and (3) the longer time between vegetables being defrosted and cooked for the cooked servings’ risk estimation (mean of 4.6 h). By contrast, in our model, not all portions are assumed to be thawed, but only 20% of them, meaning that only in such a fraction *L. monocytogenes* growth is simulated.

### 3.2. What-If Scenarios

According to the present QRA model, decreasing the *L. monocytogenes* mean concentration in pre-blanched vegetables by 1.0 log_10_ (Scenario: *μ_C_*_0_ = 0.038 log_10_ CFU/g) would generate practically the same effect as reducing the total *L. monocytogenes* cells on equipment by 2.0 log_10_ CFU in a processing lot (Scenario: *N_equip_* = 10^2^ CFU). These two scenarios would produce means of *L. monocytogenes* prevalence in packs at the end of processing of 5.0% and 5.4%—yet in very low levels of contamination (97.5th percentile of concentration of contaminated lots of 0.0024 and 0.0012 CFU/g, respectively; Table 3); which would in turn yield mean proportions of contaminated uncooked servings of 0.8% and 0.6%, respectively, and of contaminated cooked servings of 0.00148% and 0.00126%, respectively (Table 5). In both scenarios, the model estimated negligible probabilities of finding contaminated servings at the time of consumption with more than 10 CFU/g *L. monocytogenes*, even if non-RTE frozen vegetables are consumed uncooked (0.00467% and 0.00095%; Table 5).

Increasing the total *L. monocytogenes* cells on equipment in a processing lot (Scenario: *N_equip_* = 10^6^ CFU) was found to have a stronger impact on exposure than increasing the *L. monocytogenes* mean concentration in pre-blanched vegetables by 1.0 log_10_ (Scenario: *μ_C_*_0_ = 2.038 log_10_ CFU/g); as attested by the higher prevalence of contaminated uncooked servings (8.76% versus 5.45%) and cooked servings (0.08% versus 0.01%; Table 5). Higher contamination levels on equipment produce a greater spread of the microbial cells in the lot being processed, causing the microbial concentration to be not only higher on average (0.0178 CFU/g versus 0.0017 CFU/g; Table 3) but also more variable from lot to lot (97.5 percentile of 0.2345 CFU/g versus 0.0120 CFU/g; Table 3) and more variable between servings, particularly noticeable in the uncooked ones (97.5 percentile of 0.1882 CFU/g versus 0.0139 CFU/g; Table 5).

According to AFFI [50] and PROFEL [35], blanching by immersion in hot water or steam can be carried out at temperatures as low as 65 °C, subject to the type of commodity, size of pre-cut pieces, and seasonal variability. Thus, what-if scenarios with blanching temperatures (71 °C, 77 °C) lower than the one assumed for the reference scenario (83 °C) were conducted. Lowering the blanching temperature to 71 °C would produce a six-fold higher prevalence of contaminated packs at the end of processing (56.0%) in comparison with the reference scenario, with higher *L. monocytogenes* concentrations in contaminated lots (mean 0.0148 CFU/g; 97.5 percentile 0.113 CFU/g; Table 3). Correspondingly, its respective uncooked servings would have a high prevalence of contamination (20.1%), with increased concentrations of *L. monocytogenes* per serving of up to 0.124 CFU/g (97.5 percentile; Table 5). Nonetheless, it should be noticed that even though a low blanching temperature produced the highest contamination prevalence estimates among the processing-related scenarios evaluated, the numbers of *L. monocytogenes* still remained low: at the end of processing, no pack exceeded the *L. monocytogenes* concentration in frozen vegetables of 10 CFU/g; whereas, at consumption, on average 0.05% of the contaminated servings would exceed 10 CFU/g (Table 5).

The worst-case scenario illustrates a scenario of multiple co-occurring risk factors during processing—namely, a high *L. monocytogenes* concentration in pre-blanched vegetables, greater contamination on equipment post-blanching, and a milder blanching treatment. It should be kept in mind, however, that the worst-case scenario of multiple co-occurring risk factors evaluated in this study is rare because (1) the concentrations of *L. monocytogenes* in raw produce are very likely to be low and (2) normally, the cross-contamination of food from the processing environment will not result in high numbers, since *L. monocytogenes* numbers on surfaces are generally low (Zoellner et al. [8]).

In the worst-case scenario, the prevalence of contaminated packs at the end of processing was greatly increased (81.8%; Table 3), with contaminated lots attaining higher concentrations at a mean of 0.1641 CFU/g (equivalent to a mean of 82 CFU in 500 g packs from a contaminated lot). In such a rare scenario, not cooking the vegetables would be linked to a high mean risk per lot of 1.437 × 10^−11^ (Table 6), exposing the consumers to contaminated uncooked servings at a probability of 51.1%, and when contaminated, 0.50% of them would have concentrations higher than 10 CFU/g (Table 5). Yet, proper (intended) consumer handling at home (i.e., cooking) would decrease the prevalence of contaminated servings to 0.62% with very low concentration levels that hardly ever would exceed 10 CFU/g.

**Figure 2:**
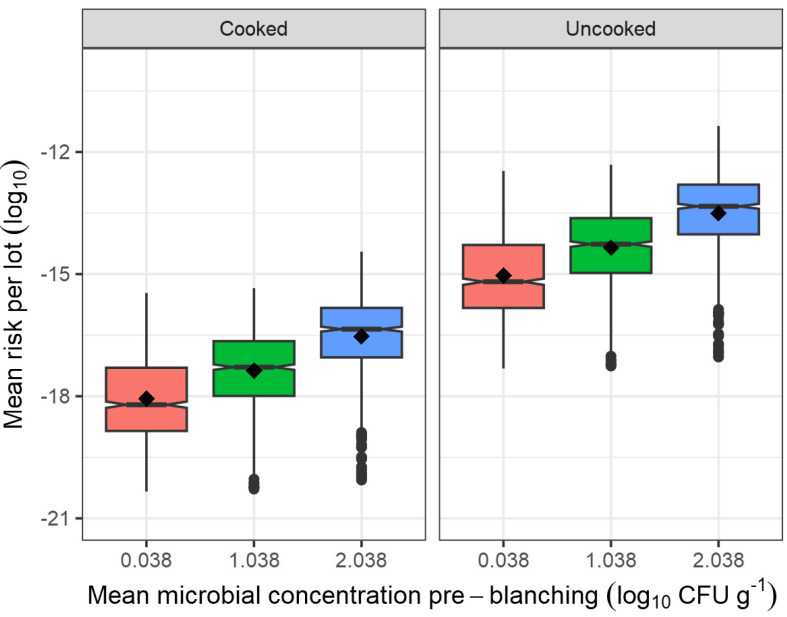
Effect of mean concentration of *L. monocytogenes* in pre-blanched vegetables and home cooking status on the mean risk of listeriosis in a serving per lot.

**Figure 3:**
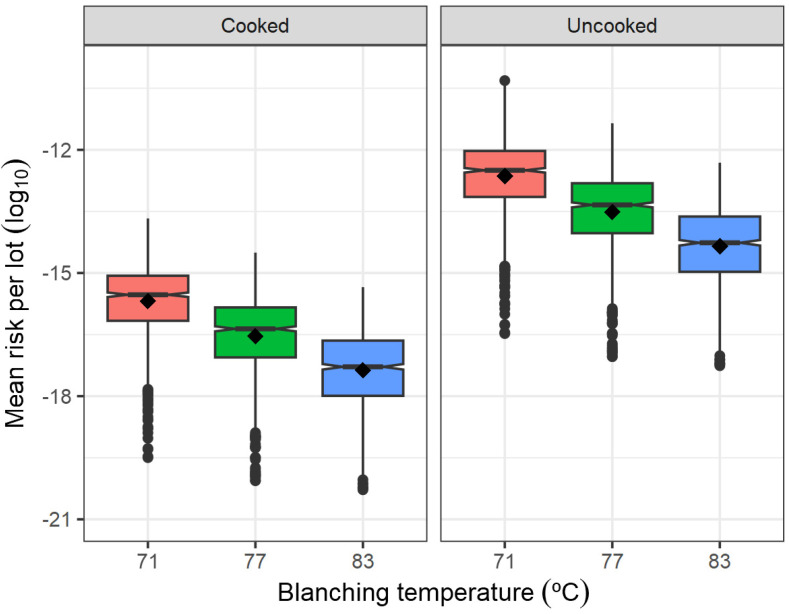
Effect of blanching temperature and home cooking status on the mean risk of listeriosis in a serving per lot.

**Figure 4:**
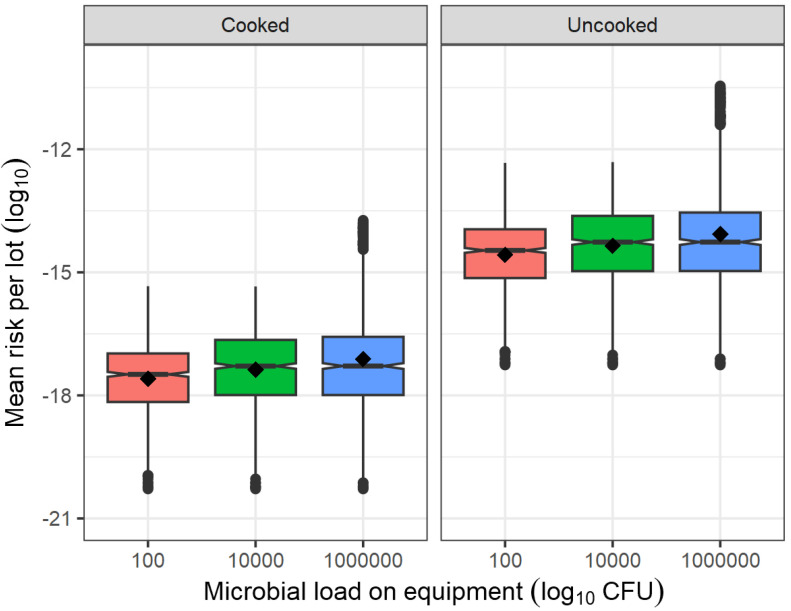
Effect of microbial load on equipment processing vegetables after blanching and home cooking status on the mean risk of listeriosis in a serving per lot.

Comparing the risk per lot estimates between the scenarios evaluated, it was found that, opposite to the mean concentration of *L. monocytogenes* in pre-blanched vegetables (Figure 2) and blanching temperature (Figure 3), changes in the microbial load on post-blanching equipment produced a very low effect on the average of the mean risk per lot and a higher effect on its between-lot variability, in particular, at the high contamination level on equipment of 10^6^ CFU (Figure 4).

Therefore, according to the present QRA model, it can be deduced that, on a batch basis, the contribution of the incoming raw produce to the final risk is greater than the contribution of re-contamination post-blanching. In environments that have been proven to harbor *L. monocytogenes* niches and lead to frequent cross-contamination, such as those of deli meats retail establishments, a QRA model [57] demonstrated that minimizing the incoming *L. monocytogenes* cells and the transfers from the environment or niches directly decreases the predicted risk of illness; and that more frequent environmental contamination events have more impact on risk than a greater number of *L. monocytogenes* cells per cross-contamination event.

Simulation results have shown the impact of the initial contamination of raw materials on the prevalence of *L. monocytogenes* in frozen vegetables at the end of processing and, consequently, the final listeriosis risk, which underlines the need for applying preventive measures of good agricultural practices. In addition, in the processing facilities, the maintenance of good hygiene conditions, including both food contact surfaces and non-food contact surfaces, cannot be sufficiently overemphasized and constitutes a strong factor that increases the likelihood of contaminated frozen vegetables, especially if cross-contamination events occur in the post-blanching processing environment such as conveyor belts and freezing equipment. The most critical steps should be considered when establishing a routine monitoring program for *L. monocytogenes* in the food processing environment of frozen vegetables. Further, sanitation standard operating procedures and corrective actions should be implemented to reduce *L. monocytogenes* contamination in the facilities. EFSA [7] has conducted an in-depth assessment of the pre-requisite program activities in the production process of blanched frozen vegetables, mainly including raw materials, infrastructure, cleaning and disinfection, water and air control, and personnel. Furthermore, EFSA [7] has provided recommendations on routine monitoring, processing environment monitoring, and end-product monitoring for processors of blanched frozen vegetables. In the same year, the European Association of Food and Vegetable Processors [35] published hygiene guidelines for the control of *L. monocytogenes* in non-RTE frozen vegetables, aiming to prevent the pathogen from colonizing and persisting in complex biofilm formation and to prevent contamination with the pathogen post thermal treatment during further handling and before packaging. PROFEL [35] details the environmental controls that need to be established to corroborate the effectiveness of the implemented pre-requisite programs and HACCP plan and to assess the potential accumulation of *L. monocytogenes* in the broader production environment.

The comparison of what-if scenarios demonstrated that the final risk was most sensitive to whether the serving was cooked or not (Figure 2, Figure 3 and Figure 4). Across all scenarios, the effect of cooking on the final risk proved to be consistent, in the order of reducing the mean risk per lot by 3.02–3.32 log_10_ (Table 6). This quantification is very pertinent in light of the increased popularity of smoothies, dips, and similar uncooked blended preparations that can be potentially elaborated from non-RTE frozen vegetables [58]. The absence of a pre-cooking step can have serious food safety consequences, as demonstrated by the *L. monocytogenes* outbreak associated with frozen corn that occurred in Europe between 2015 and 2018 and caused 53 cases and 10 deaths [7]. Therefore, frozen raw vegetables used in uncooked food preparations by consumers should not be overlooked as a source of potential foodborne outbreaks. The present model demonstrated that such an improper practice can turn a low-risk food (2.765 × 10^−17^) into a medium-risk food (2.935 × 10^−14^). Therefore, in addition to processing environment monitoring activities and end-product testing at the processing stage, consumers should be clearly informed through proper package labeling that these products are of a non-RTE nature and that thorough cooking should be ensured prior to consumption [7]. Further indications should also be given on how these products should be stored and the maximum time to be kept unfrozen in the refrigerator. Likewise, if the non-RTE frozen vegetable is intended for catering, the caterer should be instructed to ensure proper handling and adequate heat treatment during preparation. According to the reference scenario, the mean risk per serving is very low; and would consequently be the probability of observing more than one case per lot. However, this model is not contradictory with the reported outbreaks linked to these products since the implicated frozen vegetables covered long production periods and possibly involved multiple lots caused by a persistent strain belonging to the group of the most virulent strains. If we were to consider an extreme scenario of highly contaminated incoming vegetables (*μ_C_*_0_ = 2.038 log_10_ CFU/g), frequent failure to disinfect processing equipment (*N_equip_* = 10^6^ CFU, $P_{cc}$ = 0.30), milder blanching process (*Temp_Blanch_* = 68 °C, *Time_Blanch_* = 0.75 min) and unintended use in salads or smoothies (*p_Cook_* = 0), the lot-level mean risk per serving would be as high as 5.335 × 10^−8^ (median: 4.727 × 10^−9^; 95% CI: 1.244 × 10^−11^–4.002 × 10^−7^) in the elderly population (as per the dose-response model of Pouillot et al. [59]). It is, therefore, very likely that the outbreaks that occurred in the USA and Europe would have arisen from an unusual combination of failures at both processing and consumer levels that turned a regularly negligible-risk product such as frozen vegetables into a high-risk product.

In the present study, in order to illustrate the QRA model’s functionality, only a few what-if scenarios (i.e., parameters) have been evaluated. However, it is possible to obtain an in-depth assessment of the effect of multiple parameters (e.g., prevalence of *L. monocytogenes* in pre-blanch vegetables, blanching time, probability of recontamination, probability of defrosting, defrosting time/temperature, etc.) on the final risk of listeriosis, through the installation of the R package qraLm (https://github.com/WorldHealthOrganization/qraLm, accessed on 10 September 2024) containing all model’s functions and an accompanying shiny interactive dashboard.

## Figures and Tables

**Table 2:** Retrieved data on *L. monocytogenes* occurrence in minimally processed and fresh whole vegetables used for modeling the lot-to-lot variability in the pathogen’s prevalence in pre-blanched vegetables.

Country	Product	Sample Size, *n*	Positive Enrichment, *s*	Prevalence (%)	Source ^1^
*Minimally processed*					
Austria	RTE produce ^3^	143	0	0.00	Wagner et al. [26]
Italy	RTE vegetables salads ^3^	56	0	0.00	Pianetti et al. [24]
Italy	RTE vegetables ^2^	699	2	0.28	De Giusti et al. [17]
Spain	Brocolli fresh-cut ^3^	16	1	6.25	Moreno et al. [23]
Italy	Minimally processed pumpkins ^3^	33	1	3.03	Cardamone et al. [14]
Italy	RTE packed vegetables ^3^	1160	4	0.34	Losio et al. [21]
*Fresh whole vegetables*					
Italy	Vegetables ^3^	738	33	4.47	Gianfranceschi et al. [18]
Greece	Peppers ^2^	60	8	13.3	Kokkinakis et al. [19]
	Peppers ^2^	60	12	20	
Spain	Raw whole vegetables ^3^	141	2	1.41	Badosa et al. [13]
Italy	Whole vegetables ^2^	265	3	1.13	De Giusti et al. [17]
Spain	Fresh broccoli ^3^	17	2	11.7	Moreno et al. [23]
Romania	Fresh onion ^2^	10	0	0	Carp-Carare et al. [15]
Turkey	Raw vegetables ^3^	44	6	13.6	Cetinkaya et al. [16]
Albania	Onion ^3^	16	0	0	Lika et al. [20]
	Broccoli ^3^	21	0	0.00	
Czech Republic	Vegetables ^3^	249	16	6.42	Vojkovska et al. [25]
*Vegetables arriving at frozen food facility*					
USA	Corn	59	8	13.6	Magdovitz et al. [22]
	Carrots	54	0	0.00	
	Green beans	72	3	4.17	
	Peas	96	6	6.25	

^1^ Data extracted from the Pathogens-in-Foods database [12], except for [22]. In all sources, a subsample weight of 25 g was used for enrichment. ^2^ Sampled at the packinghouse. ^3^ Sampled at retail.

**Table 3:** Simulation outcomes of the exposure assessment model of *L. monocytogenes* in 500 g packs of non-RTE frozen vegetables at the end of processing for the reference and selected what-if scenarios.

Scenario	Mean Counts (CFU/g) in Contaminated Lots (Mean; Median; [95% CI])	Prevalence of Contaminated Packs	P(N > 10 CFU/g in a Contaminated Pack)	P(N > 100 CFU/g in a Contaminated Pack)
Reference (*μ_C_*_0_ = 1.038 log_10_ CFU/g; *Temp_Blanch_* = 83 °C; *N_equip_* = 10^4^ CFU)	0.0003; 5.72 × 10^−5^ [8.00 × 10^−7^–0.0025]	0.094	0	0
Initial mean contamination				
Lower (*μ_C_*_0_ = 0.038 log_10_ CFU/g)	2.12 × 10^−4^; 6.80 × 10^−6^ [4.00 × 10^−7^–0.0024]	0.050	0	0
Higher (*μ_C_*_0_ = 2.038 log_10_ CFU/g)	0.0017; 0.0005 [3.20 × 10^−6^–0.0120]	0.267	0	0
Blanching temperature				
Very low (*Temp_Blanch_* = 71 °C)	0.0148; 0.0038 [2.76 × 10^−5^–0.1129]	0.560	0	0
Low (*Temp_Blanch_* = 77 °C)	0.0016; 0.0005 [3.20 × 10^−6^–0.0117]	0.264	0	0
Recontamination				
Lower (*N_equip_* = 10^2^ CFU)	1.60 × 10^−4^; 4.14 × 10^−5^ [8.00 × 10^−7^–0.0012]	0.054	0	0
Higher (*N_equip_* = 10^6^ CFU)	0.0178; 5.72 × 10^−5^ [8.00 × 10^−7^–0.2345]	0.158	0	0
Worst-case (*μ_C_*_0_ = 2.038 log_10_ CFU/g; *Temp_Blanch_* = 71 °C; *N_equip_* = 10^6^ CFU)	0.1641; 0.0513 [0.0003–1.1550]	0.818	0.0004	0

**Table 4:** *L. monocytogenes* prevalence in minimally processed and fresh whole vegetables sampled at packinghouse or at retail.

Country	Product	Sample Size	Positive Enrichment	Prevalence (%)	Source ^1^
*End of processing*					
Spain	Frozen vegetables	906	11	1.21	Aguado et al. [54]
Poland	Frozen mixed vegetables	248	113	45.6	Pappelbaum et al. [38]
	Frozen leeks	29	0	0.00	
	Frozen onions	45	0	0.00	
	Frozen vegetables	73	17	23.3	
	Frozen corn	12	1	8.33	
	Frozen green peas	110	22	20.0	
*Retail*					
Turkey	Frozen pepper	216	0	0.00	Lee et al. [52]
Poland	Frozen vegetable mix (broccoli, carrot, green beans, peas, corn, red beans, onions, pepper, potatoes)	9100	504	5.54	Skowron et al. [55]
Spain	Frozen vegetables	1750	31	1.77	Vitas et al. [56]
Portugal	Frozen sliced green peppers	31	7	22.6	Mena et al. [51]
	Frozen sliced red peppers	33	0	0.00	
	Frozen peas	27	4	14.8	
Czech Republic	Frozen vegetables (carrots, broccoli, peas, mix, sprout)	66	0	0.00	Vojkovska et al. [25]
Multiple	Frozen vegetables (peas, carrots, corn)	43	9	20.9	Moravkova et al. [53]
	Frozen vegetables	673	69	10.3	Willis et al. [33]
Ireland	Frozen vegetables	366	21	5.73	FSAI [34]

^1^ Data extracted from the Pathogens-in-Foods database [12], except FSAI [34]. In all sources, a subsample weight of 25 g was used for enrichment.

**Table 5:** Simulation outcomes of the exposure assessment model of *L. monocytogenes* in 50 g servings of non-RTE frozen vegetables for the reference and selected what-if scenarios, as solved by the intended use (cooking) and the non-intended use (not cooking).

Scenario	Cooking	Counts (CFU/g) in Any Serving (Mean; Median; [95% CI])	Prevalence of Contaminated Servings	P(N > 10 CFU/g in a Contaminated Serving)	P(N > 100 CFU/g in a Contaminated Serving)
Reference (*μ_C_*_0_ = 1.038 log_10_ CFU/g; *Temp_Blanch_* = 83 °C; *N_equip_* = 10^4^ CFU)	No	0.0006; 6.59 × 10^−5^ [2.41 × 10^−7^–0.0030]	0.0137	4.59 × 10^−5^	0
	Yes	5.21 × 10^−7^; 9.80 × 10^−8^ [3.91 × 10^−10^–3.95 × 10^−6^]	2.61 × 10^−5^	0	0
Initial contamination					
Lower (*μ_C_*_0_ = 0.038 log_10_ CFU/g)	No	3.15 × 10^−4^; 8.79 × 10^−6^ [1.24 × 10^−7^–0.0021]	0.0080	4.67 × 10^−5^	0
	Yes	2.96 × 10^−7^; 1.16 × 10^−8^ [1.95 × 10^−10^–3.44 × 10^−6^]	1.48 × 10^−5^	0	0
Higher (*μ_C_*_0_ = 2.038 log_10_ CFU/g)	No	0.0027; 0.0005 [1.30 × 10^−6^–0.0139]	0.0545	0.0001	0
	Yes	2.54 × 10^−6^; 8.41 × 10^−7^ [2.00 × 10^−9^–175 × 10^−5^]	0.0001	0	0
Blanching temperature					
Very low (*Temp_Blanch_* = 71 °C)	No	0.0228; 0.0035 [1.32 × 10^−5^–0.1244]	0.2011	0.0005	2.98 × 10^−5^
	Yes	1.75 × 10^−5^; 5.57 × 10^−6^ [2.14 × 10^−8^–0.0001]	0.0008	0	0
Low (*Temp_Blanch_* = 77 °C)	No	0.0027; 0.0005 [1.30 × 10^−6^–0.0136]	0.0537	0.0001	0
	Yes	2.46 × 10^−6^; 8.24 × 10^−7^ [2.00 × 10^−9^–1.67 × 10^−5^]	0.0001	0	0
Recontamination					
Lower (*N_equip_* = 10^2^ CFU)	No	2.63 × 10^−4^; 4.12 × 10^−5^ [2.41 × 10^−7^–0.0015]	0.0060	9.50 × 10^−6^	0
	Yes	2.52 × 10^−7^; 6.15 × 10^−8^ [3.92 × 10^−10^–1.91 × 10^−6^]	1.26 × 10^−5^	0	0
Higher (*N_equip_* = 10^6^ CFU)	No	0.0267; 6.70 × 10^−5^ [2.41 × 10^−7^–0.1882]	0.0876	0.0017	0.0001
	Yes	1.72 × 10^−5^; 9.79 × 10^−8^ [3.91 × 10^−10^–0.0002]	0.0008	0	0
Worst-case (*μ_C_*_0_ = 2.038 log_10_ CFU/g; *Temp_Blanch_* = 71 °C; *N_equip_* = 10^6^ CFU)	No	0.2712; 0.0212 [0.0001–1.5584]	0.5110	0.0050	0.0002
	Yes	0.0001; 5.62 × 10^−5^ [2.04 × 10^−7^–0.0007]	0.0062	0	0

**Table 6:** Statistics of the mean risk of invasive listeriosis per lot of non-RTE frozen vegetables in the susceptible population for the reference and selected what-if scenarios, as solved by the intended use (cooking) and the non-intended use (not cooking). The logarithm base 10 of the mean risk reduction due to cooking is shown (log_10_RR).

Scenario	Cooking	Mean	Median	2.5 pct	97.5 pct	log_10_ RR
Reference (*μ_C_*_0_ = 1.038 log_10_ CFU/g; *Temp_Blanch_* = 83 °C; *N_equip_* = 10^4^ CFU)	No	2.935 × 10^−14^	5.446 × 10^−15^	2.183 × 10^−17^	2.187 × 10^−13^	
	Yes	2.765 × 10^−17^	5.184 × 10^−18^	2.077 × 10^−20^	2.086 × 10^−16^	3.03
Initial contamination						
Lower (*μ_C_*_0_ = 0.038 log_10_ CFU/g)	No	1.673 × 10^−14^	6.497 × 10^−16^	1.088 × 10^−17^	1.882 × 10^−13^	
	Yes	1.569 × 10^−17^	6.183 × 10^−19^	1.035 × 10^−20^	1.803 × 10^−16^	3.03
Higher (*μ_C_*_0_ = 2.038 log_10_ CFU/g)	No	1.452 × 10^−13^	4.625 × 10^−14^	1.114 × 10^−16^	1.032 × 10^−12^	
	Yes	1.347 × 10^−16^	4.444 × 10^−17^	1.059 × 10^−19^	9.267× 10^−16^	3.03
Blanching temperature						
Very low (*Temp_Blanch_* = 71 °C)	No	1.209 × 10^−12^	3.171 × 10^−13^	1.195 × 10^−15^	9.023 × 10^−12^	
	Yes	9.275 × 10^−16^	2.950 × 10^−16^	1.137 × 10^−18^	6.263 × 10^−15^	3.12
Low (*Temp_Blanch_* = 77 °C)	No	1.426 × 10^−13^	4.556 × 10^−14^	1.112 × 10^−16^	9.503 × 10^−13^	
	Yes	1.304 × 10^−16^	4.363 × 10^−17^	1.058 × 10^−19^	8.818 × 10^−16^	3.04
Recontamination						
Lower (*N_equip_* = 10^2^ CFU)	No	1.400 × 10^−14^	3.422 × 10^−15^	2.183 × 10^−17^	1.035 × 10^−13^	
	Yes	1.334 × 10^−17^	3.257 × 10^−18^	2.077 × 10^−20^	9.769 × 10^−17^	3.02
Higher (*N_equip_* = 10^6^ CFU)	No	1.418 × 10^−12^	5.466 × 10^−15^	2.183 × 10^−17^	1.809 × 10^−11^	
	Yes	9.106 × 10^−16^	5.194 × 10^−18^	2.076 × 10^−20^	1.148 × 10^−14^	3.19
Worst-case (*μ_C_*_0_ = 2.038 log_10_ CFU/g; *Temp_Blanch_* = 71 °C; *N_equip_* = 10^6^ CFU)	No	1.437 × 10^−11^	3.725 × 10^−12^	1.143 × 10^−14^	1.077 × 10^−10^	
	Yes	6.866 × 10^−15^	2.957 × 10^−15^	1.080 × 10^−17^	3.883 × 10^−14^	3.32

## Data Availability

The original contributions presented in the study are included in the article, further inquiries can be directed to the corresponding authors.
